# Development of a Site-Directed Integration Plasmid for Heterologous Gene Expression in *Mycoplasma gallisepticum*

**DOI:** 10.1371/journal.pone.0081481

**Published:** 2013-11-20

**Authors:** Isolde Nieszner, Martin Vronka, Ivana Indikova, Michael P. Szostak

**Affiliations:** Institute of Bacteriology, Mycology and Hygiene, Department of Pathobiology, University of Veterinary Medicine Vienna, Vienna, Austria; Miami University, United States of America

## Abstract

Deciphering the molecular basis of the interactions between the parasite *Mycoplasma gallisepticum* and its avian hosts suffers from the lack of genetic tools available for the pathogen. In the absence of well established methods for targeted disruption of relevant *M. gallisepticum* genes, we started to develop suicide vectors and equipped them with a short fragment of *M. gallisepticum* origin or replication (*oriC*_MG_). We failed to create a disruption vector, although by adding a further short fragment of the *M. gallisepticum tufB* upstream region we created a “Trojan horse” plasmid. This is fully integrated into the genomic DNA of *M. gallisepticum*, always at the same site, *oriC*_MG_, and is able to carry and express any gene of interest in the genetic background of *M. gallisepticum*. Successful expression of a heterologous gene was shown with the *lacZ* gene of *E. coli*. When used for gene complementation or expression of hybrid genes in *M. gallisepticum*, a site-specific combined integration/expression vector constitutes an improvement on randomly integrating transposons, which might have unexpected effects on the expression of chromosomal genes.

## Introduction

Since the first successful transformation of *Mycoplasma gallisepticum* with a transposon in 1994 by Cao et al. [1], there has been little progress in expanding the molecular tool box for this avian pathogen. *Mycoplasma gallisepticum* is the causative agent for Chronic Respiratory Disease in chickens and Infectious Sinusitis in turkeys (for review see 2) and is being continuously investigated, with the focus on vaccine development, deciphering virulence factors and the interplay of host-pathogen-interactions. Unfortunately, genetic studies in *M. gallisepticum* are hampered by the lack of suitable methods to genetically modify members of the genus *Mycoplasma*. In addition, there are obstacles to the practical handling of mycoplasmas: as members of the class *Mollicutes*, mycoplasmas are bacteria without cell walls that most often depend on an animal or plant host, thereby exhibiting a parasitic or commensal life-style. Streamlined by regressive evolution, mycoplasma genomes have undergone a drastic reduction in size and members of *Mycoplasma* are among the smallest free-living, self-replicating microorganisms. As a results of the loss of many biosynthetic pathways, mycoplasmas are absolutely dependent on many biosynthetic precursors that have to be provided by the host cells (for review see 3). In the laboratory, mycoplasmas need complex growth media and these, together with their fastidious growth characteristics, make handling the bacteria extremely difficult. The presence in *Mycoplasmas* of potent endonucleases [4,5] provides a further obstacle to the development of transformation methods or plasmid-based expression systems. For a long time only the two natural genetic mobile elements, the transposons Tn*4001* and Tn*916* [1], were available for the genetic manipulation of *M. gallisepticum*. Although the *M. gallisepticum* genome contains sequences thought to be derived from phages [6], no bacteriophage specific for *M. gallisepticum* has yet been described. 

Transformation experiments with a 2-kbp DNA fragment of the *Spiroplasma citri* chromosome, comprising the *dnaA* gene with surrounding DnaA boxes, gave the first evidence that this region is the origin of replication of *S. citri* [7]. Placed on an *E. coli* plasmid, it enabled the plasmid to autonomously replicate, even after deletion of the *dnaA* gene [7]. Similar artificial *oriC* plasmids, containing the chromosomal *dnaA* gene and surrounding DnaA box sequences, have been created for use in other mollicutes. *M. pulmonis* and three *Mycoplasma* species belonging to the *M. mycoides* cluster, *M. mycoides* subsp. *mycoides* LC (MmmLC), *M.mycoides* subsp. *mycoides* SC (MmmSC) and *M. capricolum* subsp. *capricolum* (Mccp), were successfully transformed by homologous *oriC* plasmids. In addition, the plasmids have been stably maintained as free extrachromosomal elements [8]. In Mccp an *oriC* plasmid was used to successfully express the *lacZ* gene of *E. coli* and, despite noticeable codon usage differences between *E. coli* and *Mycoplasma*, functional *E. coli* β-galactosidase (β-Gal) was detected in transformed Mccp cells [9]. 

*oriC* plasmids have also been created for the purpose of gene inactivation by homologous recombination, for example in *M. agalactiae* [10] or in Mccp [9]. Janis et al. concluded that the rare events of homologous recombination in *Mycoplasmas* were potentiated by the use of heterologous *oriC* plasmids in which the *oriC* fragment derives from a related *Mycoplasma* species. This finding was supported by the work of Lee et al. [11], who used the *oriC* region upstream of the *soj* gene of *M. gallisepticum* and its closest relative, *M. imitans*, to create plasmids that were able to replicate in both species. A targeted disruption of a *M. gallisepticum vlhA* gene was achieved with a heterologous *oriC* plasmid, while the homologous *oriC* plasmid resulted in the integration of the plasmid into the genomic *oriC* region. While transformants contained the plasmid almost exclusively as an extrachromosomal element during the first few passages, integration started to occur after 10 to 15 passages.

To develop an expression plasmid system for *M. gallisepticum*, we used a fragment of the *M. gallisepticum oriC* comprising the smallest *oriC* fragment, a 180-bp sequence, that was shown by Lee et al. to direct plasmid integration. However, this fragment extends into an additional neighbouring DnaA box. Using the 420-bp fragment in combination with a standard *E. coli* plasmid, we generated a plasmid that did not autonomously replicate in *M. gallisepticum* but instead integrated fully and almost exclusively into the *M. gallisepticum oriC* region from the first passage on. Taking advantage of this feature, we equipped the integration vector with a 203-bp fragment of the *M. gallisepticum tufB* upstream region, which we suspected to contain a promoter-like structure. We tested the integration/expression vector by subcloning the *E.coli lacZ* gene and measuring the β-galactosidase activities of the transformants. The results show the general applicability of this plasmid for site-directed gene delivery in *M. gallisepticum*. 

## Materials and Methods

### Bacterial strains and growth media

The clonal variant RCL1 [12] of *M. gallisepticum* strain R_low_ (kindly provided by S. Levisohn, Kimron Veterinary Institute, Bet Dagan, Israel) was grown at 37°C in modified Hayflick medium [13] containing 20% (vol/vol) heat-inactivated horse serum (Gibco Products, Invitrogen Ltd, Paisley, UK) (HFLX). For subcloning routines *E. coli* DH10B (LifeTechnologies GmbH, Darmstadt, Germany) was grown in Luria Bertani medium (LB). 

### Construction of Plasmids

The tentative disruption vector pDGA1-1 was created by subcloning a 2,754-bp *gapA* fragment (primers gapAFor10/gapARev11, see Table 1), amplified by PCR from genomic DNA of *M. gallisepticum* R_low_ and digested with *Xho*I, into plasmid pGEM5Zf+ (Promega, Mannheim, Germany) which was linearized by *Sal*I, taking into account that both restriction enzymes generate compatible cohesive ends. Subsequently, the *gapA* fragment was disrupted by replacing an internal 390-bp *Hin*cII fragment with a *tet*PO/*tetM* cassette generated by PCR (primers McTetFor/McTetRev) and digested with *Eco*RV, thereby creating a 5´*gapA*-*tet*PO*/tetM*-3´*gapA* gene fusion (Figure S1). Plasmid pAM120 [14] served as the template for the *tet*PO/*tetM* cassette which confers resistance to tetracycline. For creation of pDGA-oriC, a 420-bp fragment of the genomic *oriC* region of *M. gallisepticum* RCL1 was amplified by PCR (primers ori1/ori2), and subcloned via T/A cloning into pGEM-Teasy (Promega). From the resulting plasmid pGEM-oriC, the *oriC* fragment as well as a ColE1 plasmid replicon were obtained as a 2,005-bp fragment by restriction with *Sac*II and *Fsp*I, and ligated with the 4.9-kb *Sac*II/*Bsr*BI-fragment of pDGA1-1 which contained the 5´*gapA*-*tet*PO*/tetM*-3´*gapA* fragment (Figure S1). The final integration vector pINT emerged from pDGA-oriC by consecutively deleting a 2,241-bp *Age*I/*Bsp*HI (3´*gapA*) and a 779bp *Eco*RV/*Bgl*II restriction fragment (5´*gapA*). This deletion strategy removed any *gapA* sequences from the final construct.

**Table 1:** Oligonucleotide sequences.

**Primer**	**Sequence (5´to 3´)**	**Product (length [bp])**
**5TufPO**	AATCCGCGGCCTGCCATTAATTAACAAATTC	***M. gallisepticum tuf*PO (203)**
**3TufPO**	TATCCGCGGATCCATTTTTTTAAATATTTCTCC	
**ori1**	CTTTGTTGTCGATCGTAATATAAAG	***M. gallisepticum oriC* (420)**
**ori2**	ATAGAAAAACGATCGTCTATAAAC	
**McTetFor**	GATTTGATATCAGATCTGAACGGGAGTAATTGGAAG	**pAM120 *tet*PO + *tetM* (2,388)**
**McTetRev**	CTTTAGATCTGATATCATATTTATATAACAACAT	
**TetF**	catgtggagatagaac	***tetM* probe (430)**
**TetR**	gatattcctgtggcgc	
**gapAFor10**	ATATTACTCGAGGAAATGAATTCACAAGGCCAATC	***M. gallisepticum gapA*´ (2,754)**
**gapARev11**	ATTTAACTCGAGGAAGTCATTGGTTGCTCTAGAACG	
**lacZ-Bam**	ATAGGATCCATGACCATGATTACGGATTC	***E. coli lacZ* (3,149)**
**lacZ-Nar**	TTAGGCGCCTACATAATGGATTTCCTTACG	
**SDlacF**	GATCTGTTAATTAACTTTCTTTATCACACAGGAAACAGCTATGG	***E. coli lac*SD (48)**
**SDlacR**	GATCCCATAGCTGTTTCCTGTGTGATAAAGAAAGTTAATTAACA	

LacZ expression plasmids were created by subcloning first a *tuf*PO PCR product (primers 5TufPO/3TufPO), representing 203 bp of the upstream sequence of *M. gallisepticum tufB*, to pINT linearized by *Sac*II. Then, a 3.6-kb *Bam*HI fragment of plasmid pAW-lac [15], comprising the full-length *lacZ* gene of *E. coli* with its native SD_*lac*_, was inserted into the *Bam*HI restriction site behind *tuf*PO. The plasmid with *lacZ* orientated in the same direction as the *tuf*PO was designated p5TlacZ+, while p5TlacZ- carried *lacZ* in the opposite direction. For a direct fusion of *lacZ* with the first codon of *tufB*´, the SD_*lac*_ *-lacZ* fragment of p5TlacZ- was replaced by digestion with *Bam*HI and *Nar*I and insertion of a *lacZ* fragment produced by PCR amplification (primers lacZ-Bam/lacZ-Nar, template pAW-lac). Plasmid p5TSDlacZ was generated by inserting two complementary oligonucleotides (SDlacF/ SDlacR) into the *Bam*HI restriction site of p5TufPOlacZ. The disruption of the *tufB* specific reading frame was achieved by restriction of p5TlacZ+ with endonuclease *BamH*I, and filling in the staggered ends by Klenow polymerase reaction. 

### Stability assay

After transformation of *M. gallisepticum* RCL1 with plasmid pINT, ten colonies were picked randomly from HFLX agar plates containing 10µg/ml tetracycline, and inoculated in HFLX medium without tetracycline (Tet). After over night growth, the cultures were passaged by transferring an aliquot into fresh Tet-free medium. The passages of days 0, 6, 11, 15 and 20 were checked for the number of colony-forming units (CFU) by spreading dilutions in triplicates on HFLX agar plates with or without tetracycline. After a ten days incubation period, the CFU were counted using a SMZ-U stereomicroscope (Nikon Corp., Tokyo, Japan).

### Southern blot analyses

Mycoplasma genomic DNA of RCL1, and of transformants harbouring pINT was digested with *Pvu*II (Promega), restriction fragments were separated on a 0.8% agarose gel, and blotted on Hybond N+ Nitrocellulose Membranes (GE Healthcare, Freiburg, Germany) following the Neutral transfer protocol provided by the manufacturer. Hybridizations were performed using a DIG-labeled *tetM* probe and were detected using anti-DIG alkaline phosphatase antibody conjugates (Roche Diagnostics, Mannheim, Germany) and the chemoluminescent substrate CPD-Star (Roche) according to the manufacturer´s instructions. The *tetM* probe was produced by PCR amplification of pAM120 DNA using primers TetF and TetR (Table 1).

### Quantitation of β-galactosidase activity

For determination of β-Gal activities, colonies of *E. coli* DH10B transformed with a *lacZ* expression plasmid were grown in LB medium containing the appropriate antibiotic at 37°C to an optical density at 600 nm wave length (OD_600_) of 0.4 to 0.9, while *M. gallisepticum* RCL1 transformants were grown in HFLX to mid-log phase as indicated by a colour change of the growth medium from red to orange. After centrifugation of bacterial samples at 10,000 x g (5 min for *E. coli* cultures, 10 min for *M. gallisepticum* cultures), pelleted bacteria were resuspended in 1ml Z-buffer, and the LacZ activities were determined with o-nitrophenyl-β-galactopyranoside according to the method of Miller [16]. Standardization of RCL1 samples was achieved by measuring the total protein content at 562 nm after employing a bicinchoninic acid (BCA) protein assay (Pierce Kit; Thermo Fisher Scientific, Rockford, IL), while for *E. coli* samples, the cell turbidity expressed as OD_600_ values served as a correction factor in the formula for calculating the Miller units: (OD_420_ × 1000) / (OD_600 or 562_ × ml bacterial sample added × min reaction time). 

## Results and Discussion

### Creation of a *M. gallisepticum* integration vector

**Figure 1:**
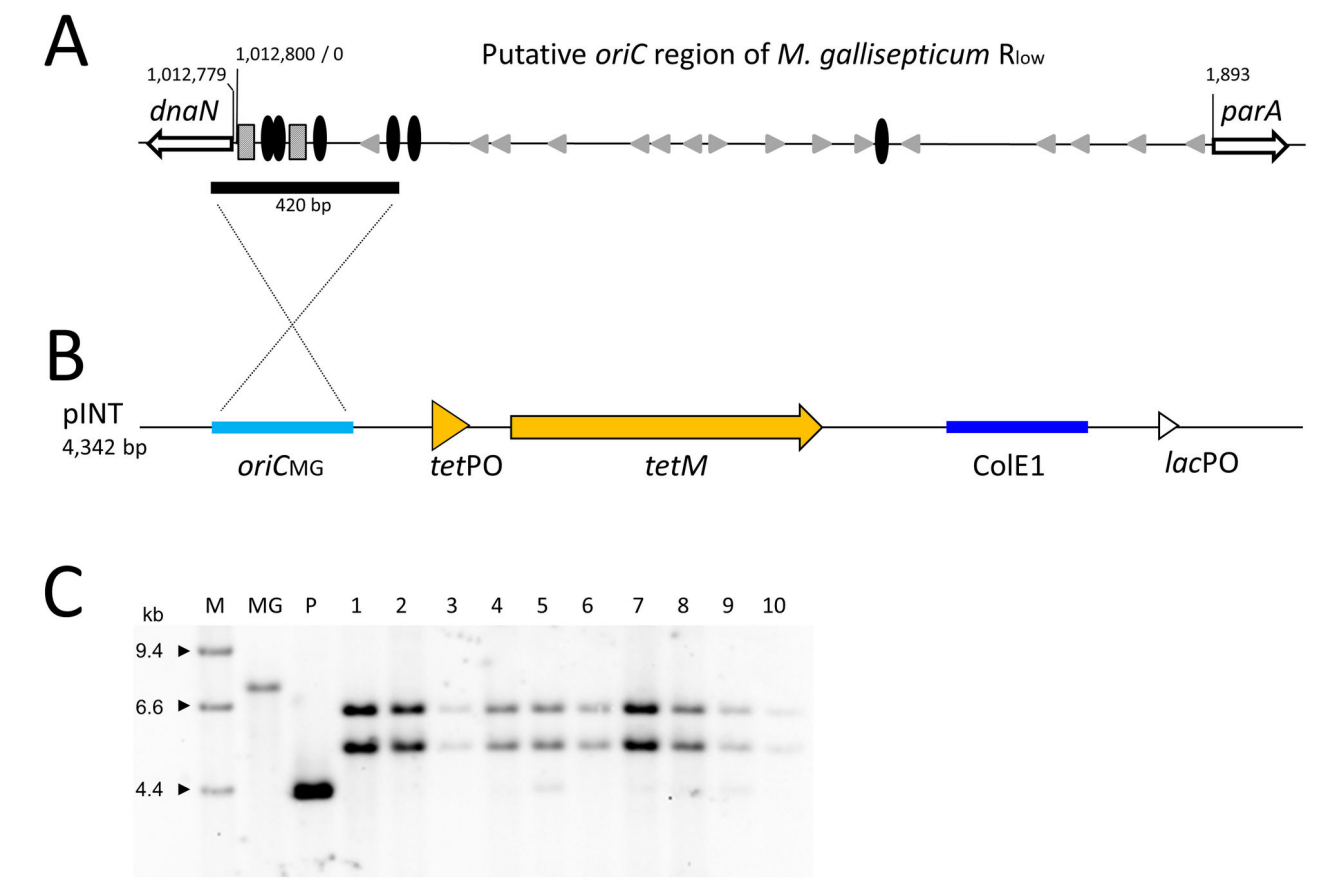
Integration of plasmid pINT into the *M. gallisepticum* genome. (**A**) Schematic representation of the origin of replication of *M. gallisepticum*. The region between *dnaN* and *soj* (parA) was annotated by Papazisi et al. to contain the origin of replication [22]. It contains DnaA boxes (black ovoids), short repeats (grey triangles), and AT-rich regions (dashed boxes). A 420-bp fragment (black bar) upstream of the *dnaN* gene enabled plasmid pINT to become integrative. Coordinates are given according to GenBank entry NC004829. (**B**) Genetic elements of plasmid pINT. ColE1, *E*. *coli* origin of replication; *lac*PO, promoter of *E*. *coli* lactose operon; *oriC*_MG_, 420-bp fragment of *M*. *gallisepticum* *oriC* region; *tetM*, tetracycline resistance gene; *tet*PO, promoter of *tetM*; (**C**) Southern blot analysis of *M. gallisepticum* RCL1 transformants harbouring pINT. Hybridization of an *oriC* probe to *Pvu*II DNA fragments of 5.6 and 6.1 kb shows the full-length integration of the 4.3-kb plasmid pINT (P, plasmid linearized by *Pvu*II) into the genomic 7.3-kb *Pvu*II fragment (MG, untransformed RCL1) which encompasses the genomic origin of replication. Ten out of 50 randomly selected Tet^R^ transformants are shown. M, DIG-labeled DNA Molecular Weight Marker II (Roche).

To create a vector for the targeted disruption of selected genes of the *M. gallisepticum* genome, the MG *gapA* gene was subcloned into pGEM5Zf and disrupted by inserting a *tetM* expression cassette, resulting in plasmid pDGA1-1. The *gapA* gene was chosen as a candidate target gene to prove the concept of integration, as natural *gapA* ^-^ mutants have already been described [12,17], demonstrating that GapA is not essential for the survival of *M. gallisepticum*. The *tetM* cassette allows screening of tetracycline-resistant (Tet^R^) mycoplasmas, which should arise from successful integration of the *gapA*::*tetM* construct by homologous recombination with the genomic *gapA* sequence. Unfortunately, our attempts to transform *M. gallisepticum* RCL1 with plasmid pDGA1-1 were not successful. To increase the probability of a homologous recombination event, pDGA1-1 was equipped with a 420-bp fragment of the *oriC* region of *M. gallisepticum*, which harbors several DnaA boxes (Figure 1A). The hope was that this would prolong the residency time of the plasmid within the bacterial cell.

Transformation with the modified plasmid, pDGA-oriC, resulted in tetracycline-resistant(Tet^R^) *M. gallisepticum* transformants, although mainly at low frequencies of about 1.8 x10^-7^ per μg plasmid DNA. To our surprise, Southern blot analyses of the transformants indicated that pDGA-oriC did not integrate into its target, the genomic *gapA*. Instead, the plasmid was found exclusively in the genomic region responsible for the origin of replication (data not shown). 

Because the tool box for *M. gallisepticum* is still limited, we decided to shape this plasmid into a general integration vector by removing the *gapA* sequences around the *tetM* gene, designating the result pINT (Figure 1B). Transformation of *M. gallisepticum* with pINT resulted in the same transformation efficiencies as seen with pDGA-oriC, and Southern blot analyses showed integration into the *oriC*_MG_ fragment in all transformants (Figure 1C). Based on size calculations it was speculated that the entire 6.9-kb plasmid could have integrated into the genomic DNA. To investigate this possibility we undertook PCR analyses with mixed primer pairs specific for plasmid and genomic sequences. The results (data not shown) confirmed that the entire pINT integrated into the genomic DNA and the sequences of the PCR products supported the notion that integration occurred via a single cross-over recombination event, initiated by the short *oriC* fragment of pINT.

Plasmid pINT seemed to become stably integrated: Tet^R^ clones of RCL1*oriC*::pINT could be grown for 20 passages on tetracycline-free liquid medium and Tet^R^ colonies were still found when the bacteria were replated on tetracycline agar plates. Determination of the CFU confirmed that even in the absence of a positive selection pressure the antibiotic resistance acquired by the integration of the pINT plasmid was not lost over a period of three weeks of continuous growth (Table 2). 

**Table 2:** Stability of pINT integration.

**Clone Tet^*R*^ clones of RCL1*oriC*::pINT [%**]*						
		**P_0_**	**P_6_**	**P_11_**	**P_15_**	**P_20_**
**1**		115	nd**	90	108	72
**2**		79	87	101	109	111
**3**		87	120	105	94	73
**4**		76	102	73	97	103
**5**		84	100	84	108	88
**6**		78	94	110	120	91
**7**		74	nd	70	101	82
**8**		108	124	90	122	124
**9**		102	104	101	89	95
**10**		122	119	nd	80	91

* Clones of RCL1*oriC*::pINT selected from a tetracycline-containing agar plate were grown for up to 20 passages in HFLX broth without antibiotic. The numbers represent the percentage of Tet**^*R*^** CFU versus whole CFU at passage number 0, 6, 11, 15, and 20.

** nd, not determined.

### Further Development into an Integrative Expression Vector

**Figure 2:**
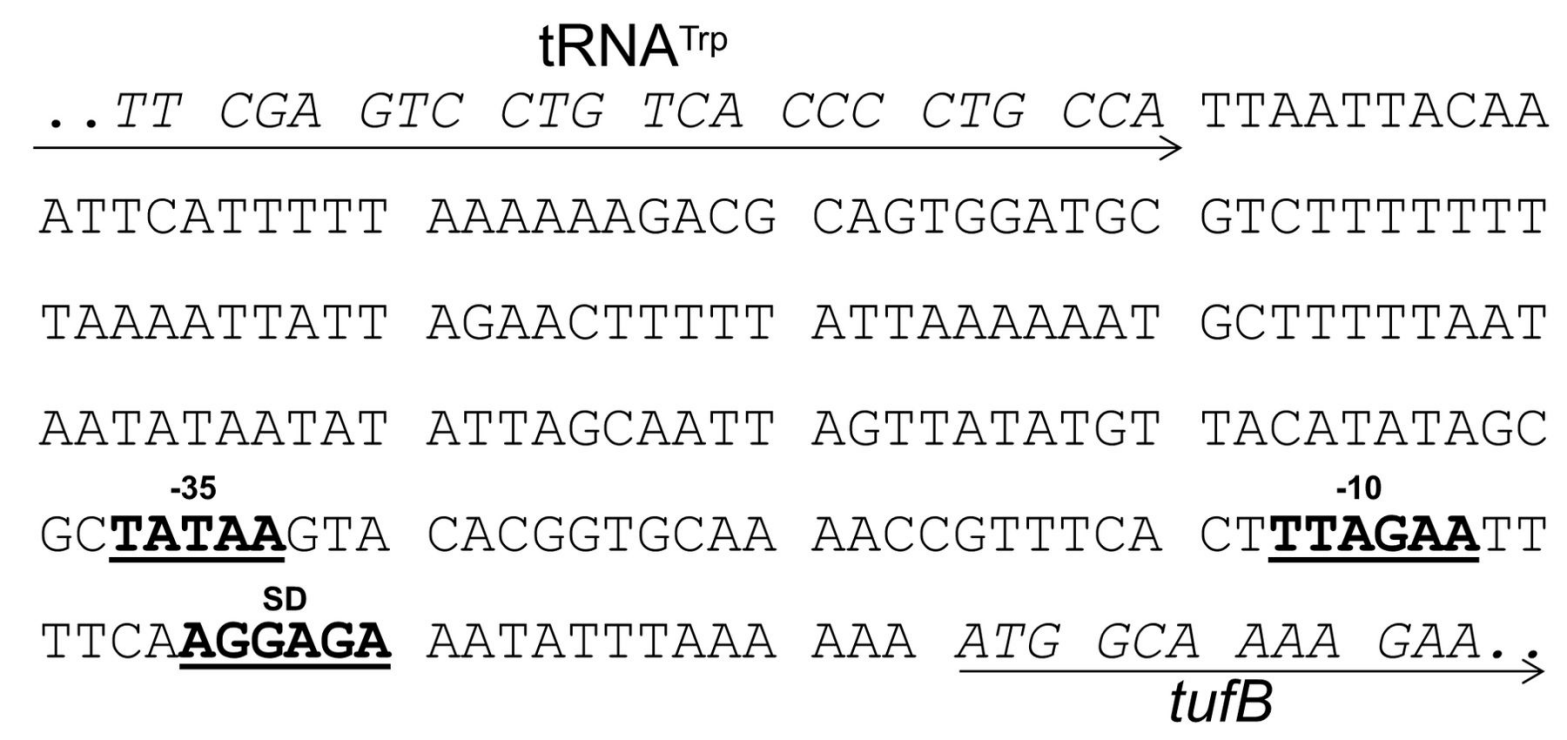
Map of the putative *M. gallisepticum* *tufB* promoter sequence. Depicted is the genomic sequence between the tRNA^Trp^ open reading frame (MGA_trna24; locus tags according to GenBank entry NC004829) and the housekeeping gene *tufB* which encodes the EF-Tu protein (MGA_1033). Arrows indicate start and end sequences of open reading frames which are additionally indicated by italicized letters, and putative transcriptional elements (-35 box, -10 box, Shine-Dalgarno sequence [SD]) are highlighted by underlined bold letters.

**Figure 3:**
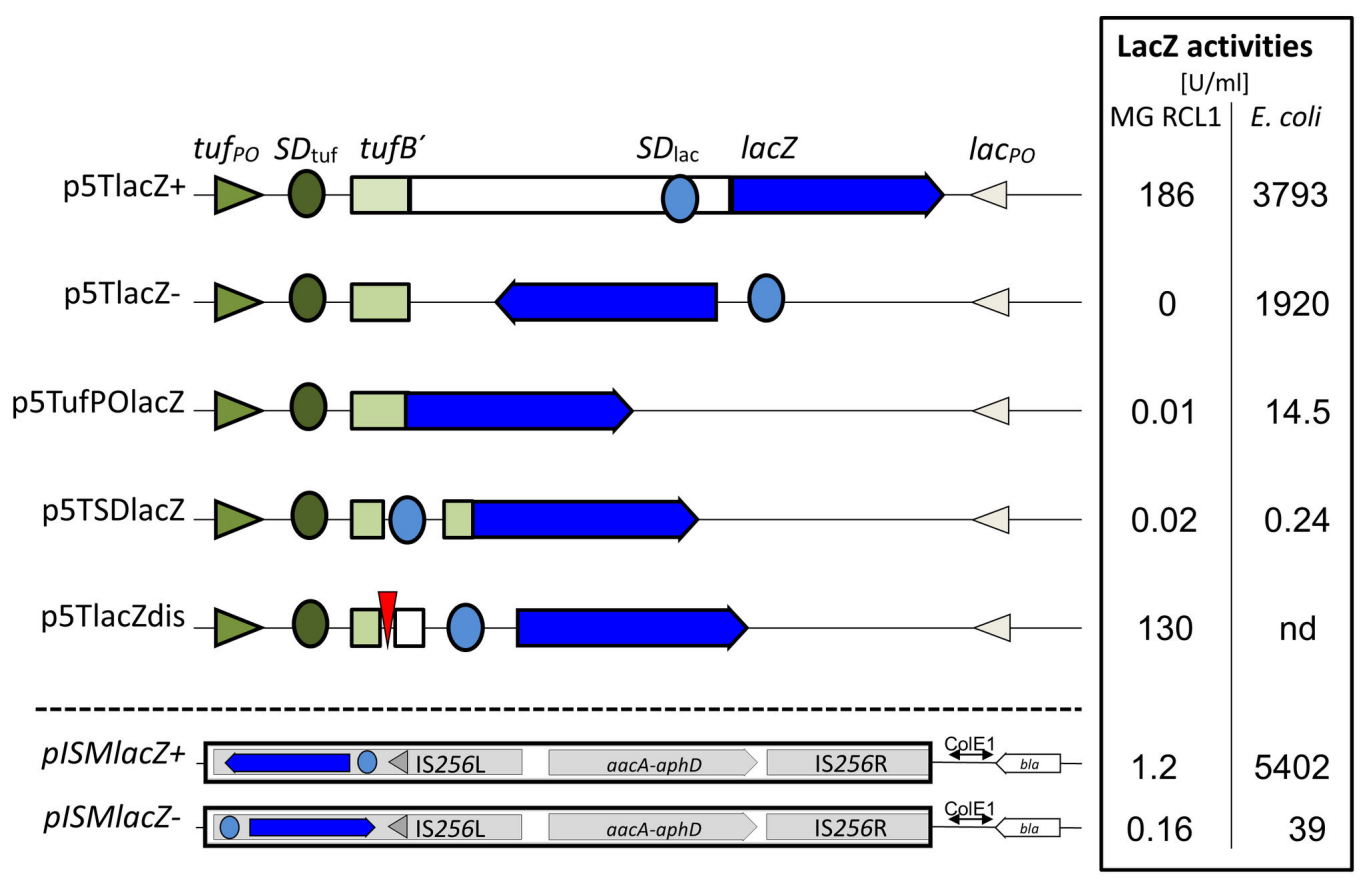
Schematical illustration of *lacZ* expression/integration vectors. The *lacZ* gene (black arrow) was subcloned with (p5TlacZ+) or without its own SD sequence (SD_lac_, black circle) (p5TufPOlacZ) downstream of a putative *tuf*PO cassette consisting of the first 4 codons of *tufB* (*tufB*`, open square), its SD sequence (SD_tuf_ , open circle), and its assumed promoter *tuf*PO (open triangle). For control reasons, plasmids were created where the SD_lac_ was inserted into *tufB*´ (p5TSDlacZ), or the *tufB* frame was terminated by creating a frame shift (p5TlacZdis), or the SD_lac_ – *lacZ* unit was subcloned in reverse orientation to *tuf*PO (p5lacZ-). For control reasons, the SD_lac_ – *lacZ* unit was also subcloned into transposon Tn*4001*mod behind the P_out_ promoter [20] (pISMlacZ+) and in opposite direction (pISMlacZ-). LacZ activities of transformed *E. coli* or *M. gallisepticum* were analyzed in 2 (*E. coli*) or 4 (*M. gallisepticum*) independent assays and are given as Miller units [16]; however, they should not be compared to each other as different methods for standardization of *E. coli* and *M. gallisepticum* samples had to be used.

To test the usefulness of pINT for heterologous gene expression, we constructed a marker gene expression cassette for the easy detection of gene expression in *M. gallisepticum*. A 203-bp fragment from the upstream sequence of the *M. gallisepticum* house-keeping gene *tufB*, encoding the EF-Tu protein, was generated by PCR and combined with the *lacZ* gene of *E. coli*, which encodes the β-galactosidase and has a long history of use as a marker gene (Figure 2) as it can produce a stable, insoluble blue compound from the chromogenic substrate X-Gal [18]. As shown by Knudtson and Minion in 1993, the *lacZ* gene is expressed in *M. gallisepticum*, where it is under the transcriptional control of a *M. gallisepticum*-specific promoter [19]. In our expression cassette, the *lacZ* gene is preceded by its own ribosome binding site (Shine-Dalgarno sequence, SD) but lacks the *lac*PO promoter. The cassette was placed between the promoter of the tetracycline resistance gene *tetM* (*tet*PO) and the *oriC* fragment of pINT, giving rise to plasmid p5TlacZ+ (Figure 3). Colonies of *M. gallisepticum* RCL1*oriC*:: p5TlacZ+ turned light blue when grown on agar plates and overlayed with X-Gal, clearly indicating that β-Gal is synthesized by the transformed mycoplasmas (not shown). Quantification of the β-Gal activities of *M. gallisepticum* RCL1 transformed with p5TlacZ+ or, as a control, with pISMlacZ+, which contains the transposon Tn*4001*mod with the same *lacZ* fragment subcloned behind the P_out_ promoter of the IS*256*L element [20] showed that LacZ expression levels were much higher in RCL1*oriC*::p5TlacZ+ (186 U/ml) than in RCL1*oriC*::pISMlacZ+ (1.2 U/ml) (Figure 3). When the *lacZ* fragment was oriented in the opposite direction to the presumptive *tufB* promoter in plasmid p5TlacZ-, no β-Gal activity (0 U/ml) was detected in RCL1 transformants. The 203-bp *tufB´* fragment thus seems to contain a transcriptionally active element that is able to promote the transcription of *lacZ* in *M. gallisepticum*. A recent publication by Panicker et al. supports this notion [21]: using a larger fragment of the *tufB* upstream region, these workers succeeded in expressing *E.coli phoA* when the gene was fused to signal and acylation sequences of the MG *vlhA*1.1 gene and placed behind the suspected *tuf*PO. 

To address the contribution of the ribosome binding site of *lacZ* (SD_lac_) or of *tufB* (SD_tuf_) for expression of *lacZ* in RCL1*oriC*::p5TlacZ+, we introduced a frame shift in the beginning of the *tufB* gene, leading to the occurrence of a stop codon 54 nucleotides downstream of the start codon. As the SD-*lacZ* fragment was unintentionally cloned in frame behind the *tufB* 5´ region, it was conceivable that we had created a *tufB*-SD-*lacZ* hybrid gene that would encode a LacZ protein with a N-terminal fusion that consists of the first five amino acids of TufB, followed by 32 unrelated amino acids. However, plasmid p5TlacZdis, with both, the disrupted *tufB* frame and the stop codon (Figure 3), was also able to establish *lacZ* expression in RCL1 transformants (130 U/ml), indicating the importance of SD_lac_ for translation. However, a readthrough of the small ribosomal subunit and reinitiation of the translation at SD_lac_ cannot be ruled out. Further supporting evidence comes from p5TufPOlacZ, where the *lacZ* fragment of p5TlacZ+ was shortened for the SD_lac_ sequence by directly fusing the *lacZ* open reading frame with the first codon of the *tufB* gene. No β-Gal activity (0.01 U/ml) was detected in RCL1*oriC*::p5TufPOlacZ transformants (Figure 3). Interestingly, no β-Gal activity (0.02 U/ml) was observed in RCL1 *oriC*::p5TSDlacZ transformants (Figure 3). Here, the SD_lac_ sequence was placed immediately upstream of the *tufB*´-*lacZ* fusion of p5TufPOlacZ to turn the non-functional p5TufPOlacZ into a *lacZ* expression plasmid. The absence of β-Gal in RCL1*oriC*::p5TSDlacZ indicates that SD_lac_ alone is not sufficient for the expression of a TufB-LacZ hybrid product (Figure 3). 

Surprisingly, p5TlacZ+, where *lacZ* is under control of the *M. gallisepticum*-derived *tuf*PO, was also able to turn *E. coli* DH10B into a *lacZ*-expressing strain, as revealed by the presence of blue colonies on X-Gal agar plates. At 3793 U per ml, the β-Gal expression levels were in the same range as those for pISMlacZ+ (5402 U/ml). The similarly high β-Gal level seen in DH10B(p5TlacZ-) (1920 U/ml) was unexpected. In this plasmid, the *lacZ* gene is oriented against the *tuf*PO. β-Gal activity can be explained by the occurrence of a remnant *lac* promoter (*lac*PO), a constituent of plasmid pGEM5Zf, which is oriented against the *tuf*PO-*lacZ* in all other plasmid constructs. As no β-Gal activity was found in RCL1*oriC*:: p5TlacZ-, the *E. coli lac*PO does not appear to be functional in *M. gallisepticum*. It should be emphasized that LacZ units given for *E. coli* and *M. gallisepticum* should not be compared to one another, as different methods were used for the standardization of samples of these diverse bacteria. Lacking cell walls, mycoplasmas do not grow as a turbid broth culture even at high numbers, so the OD_600_ of mycoplasma cells does not correlate with the number of cells, as it does in *E.coli*. For *M. gallisepticum* transformants, protein contents in samples of fresh cultures were quantified by a BCA protein assay, while *E. coli* was quantified turbidimetrically. 

The data using the modified *lacZ* expression cassettes makes it likely that *M. gallisepticum* can utilize the SD sequence of *lacZ* to express β-galactosidase encoded by the *E.coli lacZ* gene once it is placed behind a 203-bp upstream fragment of *tufB*. Attempts to create gene fusions of *lacZ* with *tufB* failed, presumably because of improper spacing of the presumed SD_tuf_ sequence to the start of the *lacZ* gene. Interestingly, the fusion of other *M. gallisepticum* genes such as *crmA* or *mgc2*, in frame with the first codon of *tufB* resulted in successful expression of CrmA or Mgc2 (data not shown). It is unclear why the expression of foreign genes in *M. gallisepticum* is more sensitive than that of autologous genes. 

In summary, we describe the successful delivery of heterologous recombinant DNA to *M. gallisepticum*. Heterologous DNA is integrated into the bacterial genome at a predetermined site and allows the synthesis of autologous and heterologous gene products in the genetic background of *M. gallisepticum*. 

## Supporting Information

Figure S1 **Construction of plasmid pDGA-oriC.** Plasmid pDGA-oriC was created by the ligation of a ColE1 / *oriC* fragment of pGEM-oriC with the *tetM*-disrupted *gapA* fragment of plasmid pDGA1-1. It should be mentioned that for creation of pGEM-oriC, a PCR fragment encoding an *oriC* fragment of *M. gallisepticum* was subcloned into pGEM-Teasy by exploiting the fact that Taq DNA polymerase exhibits terminal transferase activity. This leads to PCR fragments carrying single 3´-A overhangs at both ends, which can be fused to the 3´-T overhangs of pGEM-Teasy, often referred as T/A cloning. Another noteworthy fact is that for creation of pGA2, a PCR fragment encoding a *gapA* fragment of *M. gallisepticum* was subcloned into pGEM5Zf+ via compatible cohesive ends. Such ends were generated by cutting pGEM5Zf+ with *Sal*I, and the *gapA* PCR fragment with *Xho*I. Ligation of the compatible cohesive ends did not produce recleavable ligation products, therefore the *Sal*I and *Xho*I restriction sites are denoted in brackets. Abbreviations: *bla*, ampicillin resistance gene; *bla*PO, promoter of *bla*; *tetM*, tetracycline resistance gene; *tet*PO, promoter of *tetM*; ColE1, origin of plasmid replication; *oriC*_MG,_ origin of *M. gallisepticum* genome replication; T/A, insertion site of pGEM-Teasy for DNA fragments carrying single 3´-A overhangs at both ends.
(TIF)
