# Supplementary figures and images for: Development of a Site-Directed Integration Plasmid for Heterologous Gene Expression in Mycoplasma gallisepticum
Source: PLoS One. 2013 Nov 20;8(11):e81481. doi: 10.1371/journal.pone.0081481 (PMC3835672; doi:10.1371/journal.pone.0081481)

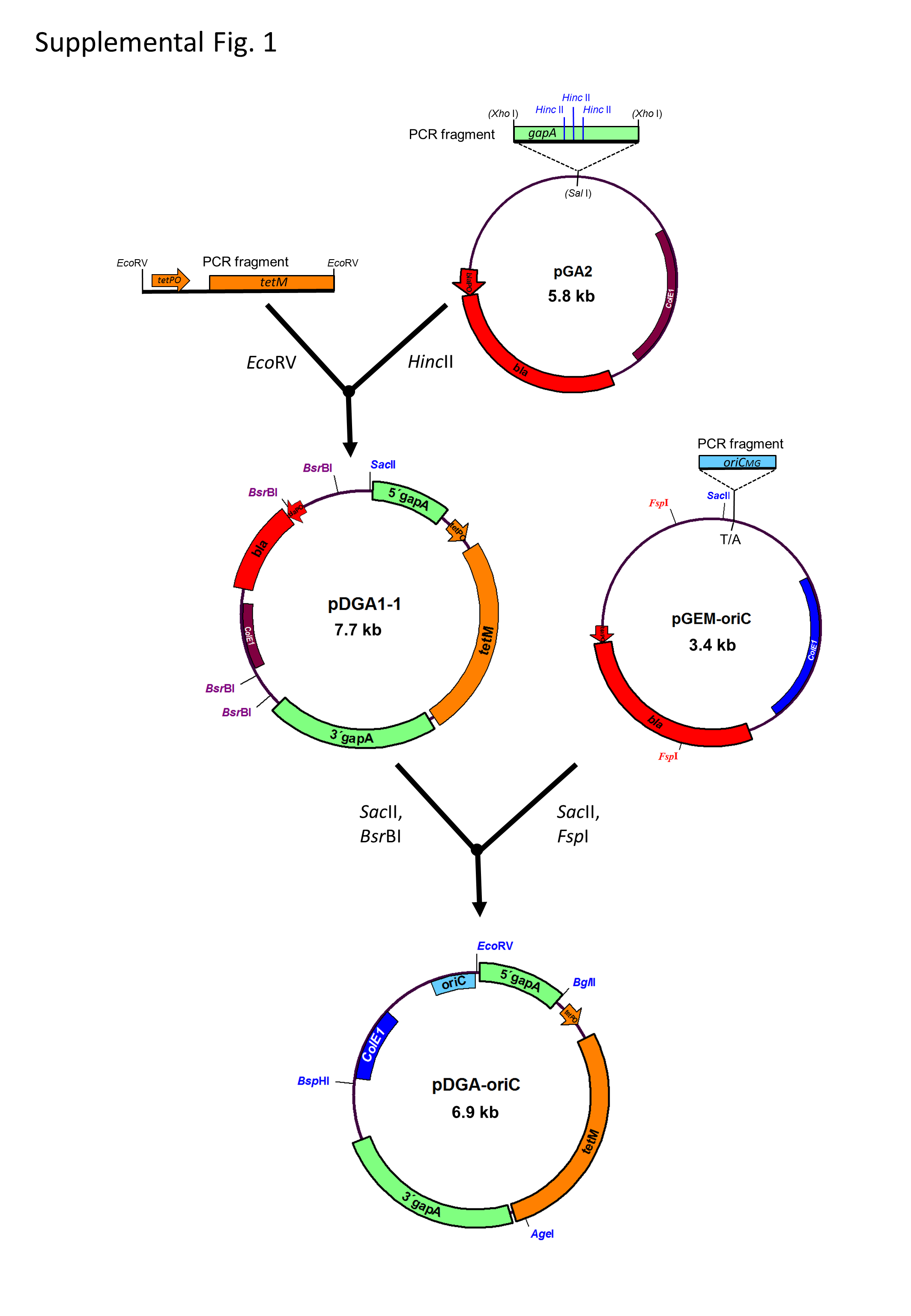

Supplement: Figure S1 — Construction of plasmid pDGA-oriC. Plasmid pDGA-oriC was created by the ligation of a ColE1 / oriC fragment of pGEM-oriC with the tetM-disrupted gapA fragment of plasmid pDGA1-1. It should be mentioned that for creation of pGEM-oriC, a PCR fragment encoding an oriC fragment of M. gallisepticum was subcloned into pGEM-Teasy by exploiting the fact that Taq DNA polymerase exhibits terminal transferase activity. This leads to PCR fragments carrying single 3´-A overhangs at both ends, which can be fused to the 3´-T overhangs of pGEM-Teasy, often referred as T/A cloning. Another noteworthy fact is that for creation of pGA2, a PCR fragment encoding a gapA fragment of M. gallisepticum was subcloned into pGEM5Zf+ via compatible cohesive ends. Such ends were generated by cutting pGEM5Zf+ with SalI, and the gapA PCR fragment with XhoI. Ligation of the compatible cohesive ends did not produce recleavable ligation products, therefore the SalI and XhoI restriction sites are denoted in brackets. Abbreviations: bla, ampicillin resistance gene; blaPO, promoter of bla; tetM, tetracycline resistance gene; tetPO, promoter of tetM; ColE1, origin of plasmid replication; oriC MG, origin of M. gallisepticum genome replication; T/A, insertion site of pGEM-Teasy for DNA fragments carrying single 3´-A overhangs at both ends. (TIF) [file pone.0081481.s001.tif]
